# An improved parameter estimation and comparison for soft tissue constitutive models containing an exponential function

**DOI:** 10.1007/s10237-017-0889-3

**Published:** 2017-03-01

**Authors:** Ankush Aggarwal

**Affiliations:** Zienkiewicz Centre for Computational Engineering, College of Engineering, Swansea University, Swansea, UK

**Keywords:** Soft tissues, Biomechanics, Constitutive laws, Nonlinear elasticity, Parameter estimation, Inverse modeling

## Abstract

Motivated by the well-known result that stiffness of soft tissue is proportional to the stress, many of the constitutive laws for soft tissues contain an exponential function. In this work, we analyze properties of the exponential function and how it affects the estimation and comparison of elastic parameters for soft tissues. In particular, we find that as a consequence of the exponential function there are lines of high covariance in the elastic parameter space. As a result, one can have widely varying mechanical parameters defining the tissue stiffness but similar effective stress–strain responses. Drawing from elementary algebra, we propose simple changes in the norm and the parameter space, which significantly improve the convergence of parameter estimation and robustness in the presence of noise. More importantly, we demonstrate that these changes improve the conditioning of the problem and provide a more robust solution in the case of heterogeneous material by reducing the chances of getting trapped in a local minima. Based upon the new insight, we also propose a transformed parameter space which will allow for rational parameter comparison and avoid misleading conclusions regarding soft tissue mechanics.

## Introduction

Formulating an accurate constitutive law for soft tissues has been a contentious research topic for several decades (Maurel et al. 1998, chap. 4). Significant advances have been made since the seminal work by Fung and others, and a multitude of hyperelastic constitutive laws have been proposed for describing the stress–strain behavior of different soft tissues. These include myocardium (Humphrey and Yin 1987), arteries (Holzapfel et al. 2000), ligaments (Natali et al. 2003; Weiss and Gardiner 2001), heart valves (May-Newman and Yin 1998).

A common feature of many of the proposed constitutive laws is the presence of an exponential function. This stems from the classic study by Fung et al. (1972), which demonstrated that stiffness of the soft tissues is proportional to stress. The exponential nature has been shown to be a result of collagen fiber recruitment and rotation that happens at the microstructural level (Lanir 1983; Billiar and Sacks 2000). However, fiber-level mechanics entails mesoscale calculations leading to high computational cost. Therefore, the phenomenological models containing an exponential are seen as better suited for tissue- or organ-scale biomechanical studies.

With a wealth of insight available on the suitability of constitutive laws, focus has been increasing on using them within biomechanical models to predict the mechanical behavior of soft tissues. Hence, accurate determination of the elastic parameters involved in the stress–strain relationships is a critical step in such predictive modeling. Various ex vivo and in vitro testing methods have been developed, and recently, methods applicable to in vivo dataset are gaining more attention as they allow the elastic properties to be characterized in tissues’ native environment.

Exponential function results in a highly nonlinear stress–strain relationship, even if we discount the geometric nonlinearity due to large deformation. In spite of this marked difference between soft tissue constitutive laws and other commonly used strain energy functions, e.g., for linear and rubber-like elastic material, standard techniques are used for parameter estimation and comparison of soft tissues. We aim to study the effect of nonlinearity of an exponential on soft tissue constitutive laws and related elastic parameters, and seek to improve upon the existing standard methods.

**Fig. 1:**
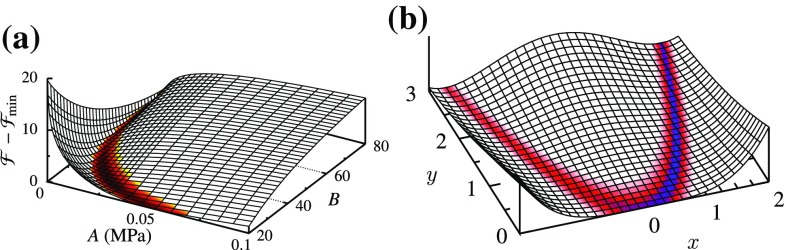
**a** Functional for the inverse modeling of bioprosthetic valve (adapted from Aggarwal and Sacks 2016) is reminiscent of (**b**) the Rosenbrock function. Only the *lowest region* is colored to emphasize their valley shape

This work is motivated by results from our recent study (Aggarwal and Sacks 2016), where an inverse model for bioprosthetic valve was developed. The study was designed to determine mechanical properties of a bioprosthetic valve leaflet by matching its deformed shape. The constitutive law contained an exponential function $\sigma \sim Ae^{B\epsilon }$ (although within a weighted integral and with neo-Hookean term), and the proposed framework included estimating two elastic parameters *A* and *B* (in the original work $c_0$ and $c_1$ were used, instead we use *A* and *B* for consistency in this manuscript). It was observed that the objective function contained a long and narrow valley in the parameter space (Fig. 1a), which resulted in a slow convergence of the inverse model, especially once the iterative solution entered the valley.

**Fig. 2:**
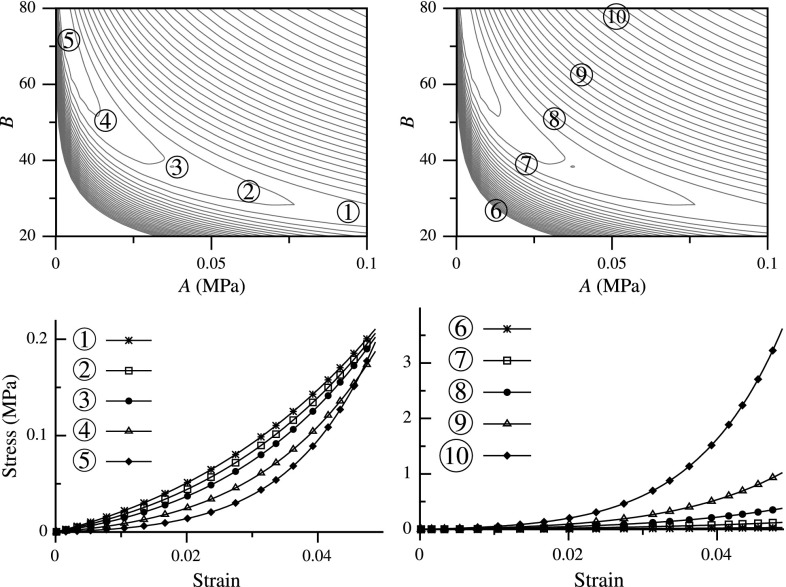
Parameters along the valley of the functional produce similar stress–strain behavior (*left*), whereas those across produce dramatically different response (*right*) (adapted from Aggarwal and Sacks 2016)

The objective function with a long narrow valley is reminiscent of Rosenbrock function used in optimization textbooks (Fig. 1b), which is a challenging minimization problem because of its shape (Rosenbrock 1960). Furthermore, the flat shape of the valley means that parameters along the valley generate closely similar stress–strain response. Thus, the two parameters *A* and *B* are highly covariant along this valley. On the other hand, changing parameters transverse to the valley dramatically affects the stress–strain response (Fig. 2). Therefore, a simple comparison of elastic parameters for tissue samples might present a grossly wrong picture. For example, parameters corresponding to points 1 and 5 are much farther apart compared to points 8 and 9 (Fig. 2). However, the former produce a much closer stress–strain response compared to the latter.

These observations raise multiple questions: did the valley shape result from that one particular problem or is it a general feature of soft tissues? Can we improve the convergence of parameter estimation? Is there a more rational way to compare the elastic parameters of two tissue samples? If only two parameters lead to a challenging parameter estimation, how will the problem behave in case of heterogeneous media (four or more parameters), where one cannot visualize the objective function? Here, we aim to answer these questions by using ideas from elementary algebra and analyzing multiple cases by extending those ideas.

## Methods and cases considered

Before starting the analysis, we present implementation details of the various functions and biomechanical models studied here. The problems were divided into two categories: displacement-controlled (DC) and force-controlled (FC). In DC cases, the input variable was the deformation or strain, and stress or forces were fitted to estimate parameters. On the other hand, in FC cases, the input variable was the force or stress, and strain or deformation were fitted.

### One-dimensional curve fitting

Starting in one dimension, the simplest function with an exponential is

1 $$\begin{aligned} \sigma (A,B,\epsilon )=Ae^{B\epsilon }. \end{aligned}$$

Here $\epsilon$ represents strain and is the input, while $\sigma$ is the output representing stress. Thus, (1) is a DC case, and the inverse of this relationship

2 $$\begin{aligned} \epsilon (A,B,\sigma )=\frac{\log (\sigma /A)}{B} \end{aligned}$$

is an FC case. Since the stress function (1) is not zero at $\epsilon =0$, we also considered a more realistic representation of stress

3 $$\begin{aligned} \sigma (A,B,\epsilon )&=A\left( e^{B\epsilon }-1\right) \text { and its inverse} \end{aligned}$$

4 $$\begin{aligned} \epsilon (A,B,\sigma )&=\frac{\log (\sigma /A+1)}{B}. \end{aligned}$$

We considered a model with higher nonlinearity:

5 $$\begin{aligned} \sigma (A,B,\epsilon )=A\left( e^{B\epsilon ^2}-1 \right) . \end{aligned}$$

Lastly, the exponential function is sometimes truncated and linearized beyond an upper bound on strain $\epsilon _\mathrm{ub}$ (Fan and Sacks 2014). This may represent the condition when all the fibers have been recruited, thus producing a linear stress–strain response thereafter. Accordingly, we considered the following model

6 $$\begin{aligned}&\sigma (A,B,\epsilon )\nonumber \\&\;\;=\left\{ \begin{array}{ll} A(e^{B\epsilon }-1) &{}\quad \text { for } \epsilon \le \epsilon _\mathrm{ub} \\ A \left[ (e^{B\epsilon _\mathrm{ub}}-1) + Be^{B\epsilon _\mathrm{ub}}(\epsilon -\epsilon _\mathrm{ub}) \right] &{}\quad \text { for } \epsilon >\epsilon _\mathrm{ub} \end{array} \right. \nonumber \\ \end{aligned}$$

The stress function (6) was defined such that both stress $\sigma$ and stiffness $\partial \sigma /\partial \epsilon$ remain continuous at $\epsilon =\epsilon _\mathrm{ub}$. In all one-dimensional problems, the input was used to calculate output which was then matched to observed data in order to obtain the optimum parameters *A* and *B*. We call this process “curve fitting.”

### Multi-dimensional curve fitting

Hyperelastic constitutive laws for soft tissues are generally defined in two or three dimensions, based upon deformation gradient $\mathbf {F}$ (Holzapfel 2000). We studied two commonly used constitutive laws here; however, the results are expected to be extensible to most others. First is the Gasser–Ogden–Holzapfel (GOH) model (Gasser et al. 2006):

7 $$\begin{aligned} \Psi (A,B,\mathbf {F})=\frac{A}{2B}\left( e^{\textit{BQ}} -1\right) + \Psi _{\text {matrix}}(\mathbf {F}), \end{aligned}$$

where $Q={\left( \kappa I_1+(1-3\kappa )I_4-1 \right) ^2}, I_1={{\mathrm{tr}}}(\mathbf {C})$ is the first invariant of $\mathbf {C}=\mathbf {F}^T\mathbf {F}, I_4=\mathbf {C}:\varvec{N}\otimes \varvec{N}$ is the fourth invariant with material direction vector $\varvec{N}$, and $\kappa$ controls the “degree of anisotropy.” $\Psi _{\text {matrix}}$ represents the contribution from the ground matrix in the tissue, and it is modeled as a compressible neo-Hookean solid:

8 $$\begin{aligned} \Psi _{\text {matrix}}(\mathbf {F}){=} \frac{\upmu }{2} \left( I_1-3 \right) {-}\upmu \log (J) {+} \frac{\lambda }{2} \left( \log (J) \right) ^2, \end{aligned}$$

where $J=\sqrt{\det \left( \mathbf {C} \right) }$ represents the volume change.

Second is the simplified structural model (SM) (Fan and Sacks 2014):

9 $$\begin{aligned} \Psi (A,B,\mathbf {F})&= \int \limits _\theta \Gamma \left( \theta \right) A \left( \frac{e^{B \left( \varvec{N}\cdot \mathbf {E}\cdot \varvec{N} \right) }-1}{B} {-} \varvec{N}\cdot \mathbf {E}\cdot \varvec{N} \right) \; \mathrm{d}\theta \nonumber \\&\quad + \Psi _{\text {matrix}}(\mathbf {F}), \end{aligned}$$

where $\mathbf {E}=\frac{1}{2}\left( \mathbf {C}-\mathbf {I} \right)$ and $\Gamma (\theta )$ is the fiber orientation function. $\Gamma (\theta )$ is modeled as a truncated Gaussian in angle $\theta \in \left[ -\pi /2,\pi /2\right]$ with standard deviation (SD) $\tau$ and peak at $\theta =\omega$:

10 $$\begin{aligned} \Gamma (\theta )=d_e\frac{1}{P}\exp \left( -\frac{(\theta -\omega )^2}{2\tau ^2} \right) + \frac{(1-d_e)}{\pi }, \end{aligned}$$

where $P=\int \limits _{-\pi /2}^{\pi /2} \exp \left( -\frac{(\theta -\omega )^2}{2\tau ^2} \right) \;\mathrm{d}\theta$ normalizes the distribution.

If we assume that microstructural parameters, such as $\kappa , \varvec{N}, d_e, \omega$ and $\tau$, and ground matrix properties are known, both constitutive models (7) and (9) have only two elastic parameters *A* and *B* to be determined. Even an approximation of the ground matrix elastic properties provides a good estimate of the overall mechanical behavior for many cases, such as bioprosthetic valves (Aggarwal and Sacks 2016). For both models, second Piola–Kirchhoff stress can be derived by the standard relation $\mathbf {S} = 2\frac{\partial \Psi }{\partial \mathbf {C}}$, and it is easy to see that $\mathbf {S}\sim Ae^{B(\cdot )}$. In (7), the exponent is *BQ*, whereas in (9), we have an integral of exponentials. Thus, the two models represent two distinct classes of constitutive laws.

GOH (7) and SM (9) models were used in multi-dimensional curve fitting, where known deformation gradients were used to calculate the stresses. These stresses were then matched to the observed stresses in order to determine the elastic parameters. Since the known input was deformation and stress was the output, these curve fitting problems belong to the DC category. We note that for these constitutive laws, there is no closed form solution of the inverse relation, i.e., strain or deformation gradient as a function of stress. Therefore, multi-dimensional curve fitting could not be performed for the FC case.

### Inverse models

For cases where an explicit relation from input to output is not available (such as the FC case for multi-dimensional problems) or where the input parameters vary spatially and cannot be represented using a single set of values, curve fitting cannot be performed. In such situations, it is more appropriate to solve an inverse model, where the observations are matched to the outcome of a finite element simulation. This finite element model uses a predetermined constitutive law, and we tested both GOH (7) and SM (9) models.

**Fig. 3:**
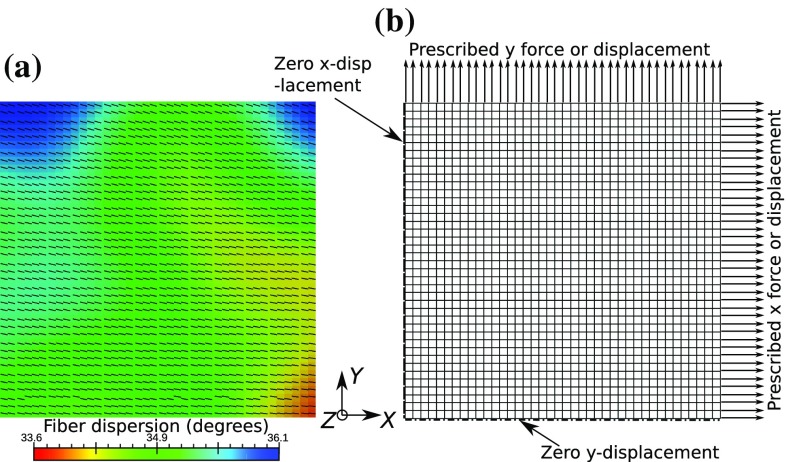
Simulation setup of the planar biaxial stretching of a square tissue sample, **a** variable microstructure and **b** described boundary/load conditions on a quadrilateral mesh

We considered two inverse modeling problems, and all of the finite element simulations were performed using FEBio (Maas et al. 2012). First problem is that of a biaxial testing of a thin planar tissue sample (Fig. 3), which was studied for both DC and FC cases. In the DC case, known uniform displacement boundary conditions were applied on the sample edge, and total reaction forces on the edges were matched to the observed values. On the other hand, in the FC case, known uniform forces were applied on the sample edge, and average edge displacements were matched to observed values. A detailed description of the setup, such as the sample size, boundary conditions, is specified in “Details of biaxial simulation.”

**Fig. 4:**
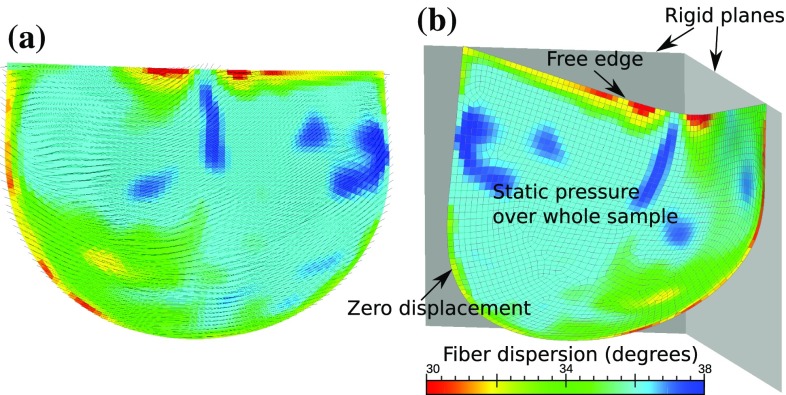
Simulation setup of a semilunar-shaped tissue sample with **a** variable fiber direction, **b** under static pressure load and in contact with two rigid planes with zero displacement boundary condition prescribed on the rounded edge

The second problem studied here is the shape matching of a semilunar tissue sample under static pressure loading (Fig. 4), which represents the closing of a bioprosthetic valve leaflet. Since the input for this problem is pressure traction, only FC case was possible. Details of the shape matching procedure were described in our previous work (Aggarwal and Sacks 2016) and are summarized in “Details of tissue pressurization simulation.” All of the problems considered in this study are summarized in Table 1.

**Table 1:** Summary of all problems considered, indicating force-controlled (FC) and displacement-controlled (DC) cases

Problem	Model	Input	Output	Type
1D curve fitting	(1)	Strain $\epsilon$	Stress $\sigma$	DC
	(3)	Strain $\epsilon$	Stress $\sigma$	DC
	(5)	Strain $\epsilon$	Stress $\sigma$	DC
	(6)	Strain $\epsilon$	Stress $\sigma$	DC
	(2)	Stress $\sigma$	Strain $\epsilon$	FC
	(4)	Stress $\sigma$	Strain $\epsilon$	FC
Multi-D curve fitting	(9)	Deformation gradient $\mathbf {F}$	$2{\text {nd}}$ PK Stress $\mathbf {S}$	DC
	(7)	Deformation gradient $\mathbf {F}$	$2{\text {nd}}$ PK Stress $\mathbf {S}$	DC
Inverse modeling of biaxial setup	SM (9)	Displacement boundary conditions	Edge forces	DC
	GOH (7)	Displacement boundary conditions	Edge forces	DC
	SM (9)	Force on edges	Edge deformation	FC
	GOH (7)	Force on edges	Edge deformation	FC
Inverse modeling of shape matching	SM (9)	Pressure	Deformed shape	FC
	GOH (7)	Pressure	Deformed shape	FC

### Generic notation

For all the cases considered, both DC and FC, we define a generic notation for the analysis. $\bar{f}(x_i)$ denotes the observed data at independent input variable $x_i$ for $i=1\ldots N$, and $f(A,B,x_i)$ denotes the model. *A* and *B* are the parameters to be determined by fitting $f(A,B,x_i)$ to $\bar{f}(x_i)$. In the present context of soft tissue mechanics *x* denotes the input applied during testing, e.g., applied strain, displacement or load, $\bar{f}$ represents the observed output, e.g., stress, force, deformed shape or strain, and *f*(*A*, *B*, *x*) is the same quantity computed using our model. Since we are interested in functions of the form $f(A,B,x)\sim Ae^{h(B,x)}$ or its inverse, *A* has units of stress and *B* is dimensionless.

To fit our model to the observed data, we define a functional $\mathcal {F}={||}f - \bar{f}{||}$. The minimum point of this functional corresponds to the optimum parameters $\breve{A}, \breve{B} = {{\mathrm{arg\,min}}}_{A,B} \mathcal {F}$, where *f* and $\bar{f}$ are “closest.” We use the norm ${||}\cdot {||}$ in a general sense, and two options were explored:

11 $$\begin{aligned} \text {2-norm: }\mathcal {F}&= {||}f - \bar{f}{||}_2 \end{aligned}$$

12 $$\begin{aligned} \text {log-norm: }\mathcal {F}&= {||}f - \bar{f}{||}_{\log } = {||}\overline{\log }(f) - \overline{\log }(\bar{f}){||}_2 \end{aligned}$$

The 2-norm is the standard Euclidean norm, which $=\sum _i \big (f(x_i) - \bar{f}(x_i) \big )^2$ for discrete input $x_i$ or $=\int _0^X \big (f(x) - \bar{f}(x) \big )^2\;\mathrm{d}x$ for continuous input $x\in (0,X)$. The “log-norm” uses logarithm of *f* and $\bar{f}$ in the standard Euclidean norm. The logarithm function is denoted as $\overline{\log }$ to clarify its modified form

13 $$\begin{aligned} \overline{\log }(x)=\left\{ \begin{array}{cc} \log (x) &{}\quad \text {if } x>0 \\ x &{}\quad \text {if }x\le 0 \end{array} \right. . \end{aligned}$$

In general, there could be constraints on *A* and *B* in our minimization problem. These constraints are physically motivated, for example from thermodynamics of strain energy density and convexity requirements for stress function. Here, we considered two constraints that both *A* and *B* must be positive.

In order to exclude the effect of noise, the observed data were generated synthetically for known values of the parameters $\bar{A}$ and $\bar{B}$. Hence, we denote the observed data as $\bar{f}(x)=f(\bar{A},\bar{B},x)$, where $\bar{A}$ and $\bar{B}$ are *known a priori*. Clearly, in this case, the global minimum of $\mathcal {F}$ should occur at $\breve{A}=\bar{A}$ and $\breve{B}=\bar{B}$. All the parameters to be determined are collectively denoted as $\mathbf {c}$, and the model *f* and data $\bar{f}$ at all inputs $x_i$ combined into a vector are denoted as $\mathbf {f}$ and $\overline{\mathbf {f}}$, respectively.

Lastly, we define two curves in the (*A*, *B*) parameter space where one of the first derivatives of the functional $\mathcal {F}$ vanishes:

14a $$\begin{aligned} A^{\text {min}}_1(B):\mathop {=}\limits ^{\text {def}} \left. \frac{\partial \mathcal {F}(A,B)}{\partial A}\right| _{A^{\text {min}}_1(B),B}&= 0 \;\; \text { and}\end{aligned}$$

14b $$\begin{aligned} A^{\text {min}}_2(B):\mathop {=}\limits ^{\text {def}} \left. \frac{\partial \mathcal {F}(A,B)}{\partial B}\right| _{A^{\text {min}}_2(B),B}&= 0 . \end{aligned}$$

We name them *A*-partial minima (APM) and *B*-partial minima (BPM) curves, respectively, or PM curves collectively. The two PM curves intersect at the global minimum $(\breve{A}, \breve{B})$, and the shape and adjacency of the two curves help develop an intuitive and qualitative understanding of the analysis. Whenever possible, closed form expressions were evaluated for PM curves. For other problems, the PM curves were determined numerically. For APM, this was done by fixing *B* at various values and minimizing $\mathcal {F}$ with respect to *A*, and vice versa for BPM.

### Parameter estimation

In order to calculate the elastic parameters for soft tissues, a numerical optimization has to be performed to minimize the function $\mathcal {F}(\mathbf {c})$. We focus on gradient-based line search methods, which proceed iteratively in two steps: (1) determine a direction along which $\mathcal {F}$ will decrease and (2) determine the step size to move along that direction (also known as line search). For calculating the direction in step 1, the simplest choice is the steepest descent direction $-\nabla \mathcal {F}$. However, it leads to extremely slow convergence for functionals with a narrow valley, such as the Rosenbrock function (Nocedal and Wright 2006). On the other hand, using the full Newton’s method

15 $$\begin{aligned} \left( \nabla ^2\mathcal {F}\right) \Delta \mathbf {c}=-\nabla \mathcal {F} \end{aligned}$$

gives second-order convergence. However, it requires calculation of second derivatives for the Hessian $\nabla ^2\mathcal {F}$ and solving a linear system of equations (15), making it computationally expensive. For least-square functionals, such as the 2- and log-norms (11,12), their form allows a simpler approximation of the Hessian. If $\mathbf {J}=\partial \mathbf {f} / \partial \mathbf {c}$, then the functional gradient is $\nabla \mathcal {F}=\mathbf {J}^T(\overline{\mathbf {f}}-\mathbf {f})$ and the first-order approximation of the Hessian is $\nabla ^2 \mathcal {F} \approx \mathbf {J}^T\mathbf {J}$. This approximation leads to the Gauss–Newton algorithm

16 $$\begin{aligned} (\mathbf {J}^T\mathbf {J})\Delta \mathbf {c}=\mathbf {J}^T(\mathbf {f}-\overline{\mathbf {f}}), \end{aligned}$$

which gives approximately second-order convergence and requires only the first derivative to be computed.

Comparing the different gradient-based methods for fitting the one-dimensional exponential function (1), we found that the Gauss–Newton algorithm performed the best. This is consistent with observations about the Rosenbrock function (Nocedal and Wright 2006). Henceforth, we used the Gauss–Newton algorithm (Algorithm 1) for all problems. For the line search in step 2, we used a simple backtracking algorithm, which took into account any constraints on the parameters and situations of failed $\mathbf {f}$ calculation (e.g., due to non-convergence of the finite element solver).
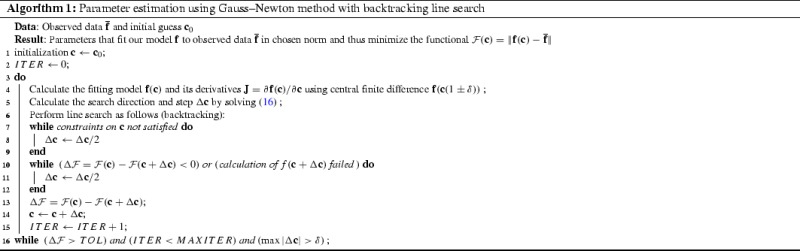


Values of $\hbox {TOL}=10^{-10}$ and $\delta =10^{-5}$ were used for all calculations. Convergence of nonlinear minimization can strongly depend on the initial guess or the starting point (denoted using subscript 0, $\mathbf {c}_0$, etc.). Therefore, for each problem, multiple minimizations were performed with starting points spanning the parameter space. Hence, $10\times 10$ starting guesses were chosen uniformly distributed in the span of $A_0\in [0.005,0.1]$ and $B_0\in [20,100]$. The resulting convergence statistics—number of iterations (#Iter), number of $\mathbf {f}(\mathbf {c})$ evaluations (#Eval) and number of parallel evaluations (#Paral)—were reported as mean ± SD. The number of parallel evaluations was important since the central difference calculations for determining $\mathbf {J}$ were done in parallel. However, during the line search, evaluations had to be performed sequentially, adding to the computational cost.

### Noise

In order to study the effect of noise on the accuracy of the estimated parameters, we added random noise of varying magnitude to the target vector:

17 $$\begin{aligned} \bar{f}(x)=f(\bar{A},\bar{B},x) \left( 1+\nu \,\text {rand}\left[ -1,1\right] \right) . \end{aligned}$$

Here $\nu$ represents the noise level, which was varied from 0.01 to 0.04, and the random number between $-1$ and 1 had uniform probability. The effect of the noise was quantified by an error in the estimated optimum parameters:

18 $$\begin{aligned} e(\nu )=\sqrt{ \left( \bar{A}-\breve{A}\right) ^2+\left( \bar{B}-\breve{B}\right) ^2 }. \end{aligned}$$

Clearly, $e(0)=0$ since without noise the global minimum coincides with the true minimum.

### Heterogeneous model

**Fig. 5:**
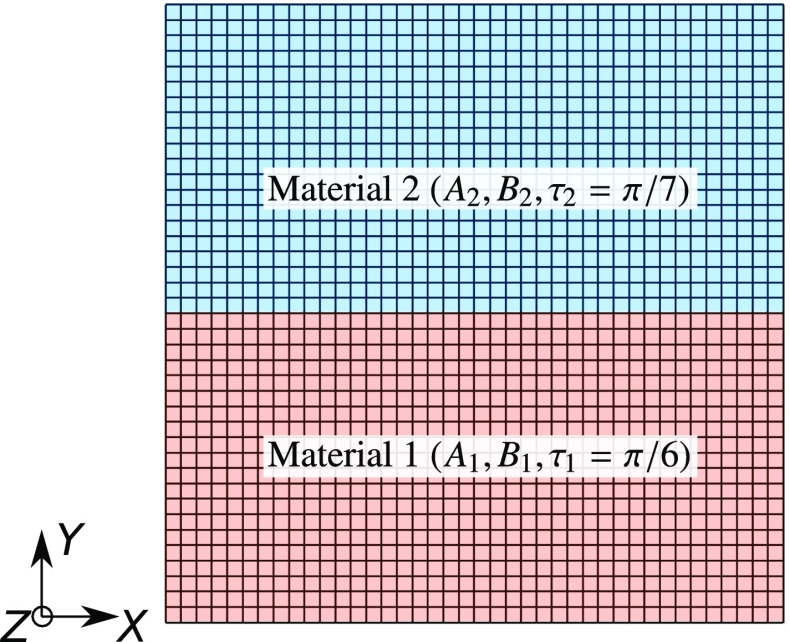
For the heterogeneous model example, consider the situation where tissue sample is made up two materials—1 and 2 with elastic parameters $(A_1,B_1)$ and $(A_2,B_2)$, respectively. $\tau _1\ne \tau _2$ is required to break the symmetry of the problem and make its Hessian non-singular everywhere

In all of the problems considered so far, we assumed that the elastic parameters did not vary over the tissue sample. However, heterogeneity is a common feature of biomechanical systems. In order to study the parameter estimation properties for a heterogeneous system, we considered the simplest problem of two materials in a biaxial testing setup. That is, the tissue sample is made of two types of tissues with two sets of elastic parameters—$(A_1,B_1)$ and $(A_2,B_2)$ (Fig. 5). The boundary and loading conditions remained the same as in the biaxial inverse model (“Details of biaxial simulation”). We considered only the SM constitutive law for both DC and FC cases. If both tissues in the sample have the same microstructural properties, then the system is symmetric, i.e., both $(A_1,B_1,A_2,B_2)$ and $(A_2,B_2,A_1,B_1)$ give exactly the same response. This leads to a singular Hessian whenever $A_1=A_2$ and $B_1=B_2$. Therefore, in order to break this symmetry and make the Hessian non-singular, we used $\tau _1=\pi /6$ and $\tau _2=\pi /7$.

In total, there are four unknown elastic parameters—$A_1, B_1, A_2$ and $B_2$, which leads to a four-dimensional (4D) parameter space. As a consequence, the functional cannot be visualized and scanning the entire parameter space for starting points becomes prohibitively expensive. Therefore, we only scanned a diagonal plane in that 4D space, where the starting parameters were the same for the two materials: $A_{1,0}=A_{2,0}$ and $B_{1,0}=B_{2,0}$. Hence, $8\times 8$ starting guess points were chosen which were uniformly distributed in the span of $\log (A_{1,0})\in [-4,-2]$ and $B_{1,0}\in [20,100]$. The parameter estimation was performed *without* adding any noise to the system.

## Results

### Analysis

Before carrying out parameter estimation, we analyze the properties of various models described in the previous section and the functional $\mathcal {F}$ constructed for them. The analysis is divided into DC and FC cases.

#### Displacement-controlled cases

**Fig. 6:**
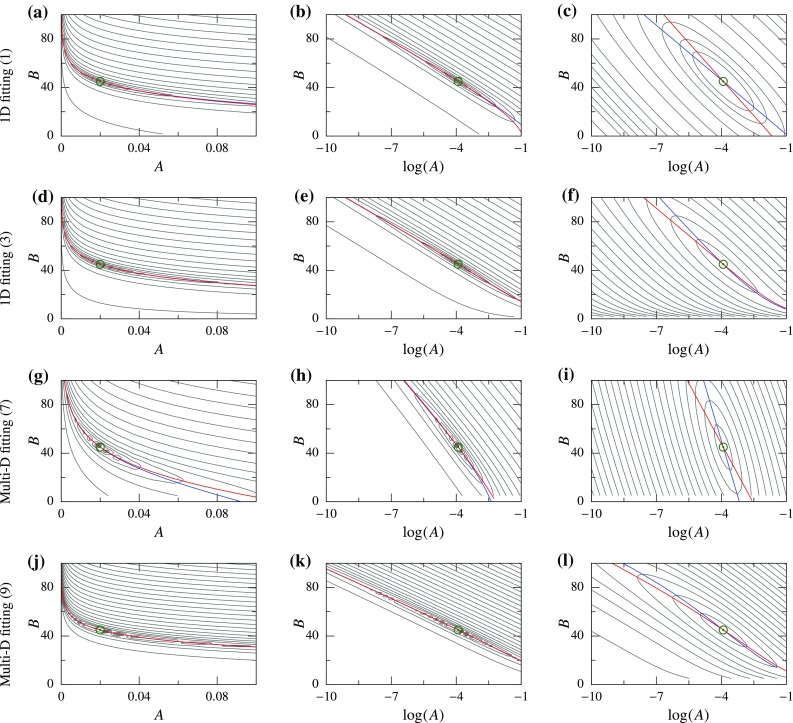
Functional for displacement-controlled (DC) cases plotted as a *contour*; global minimum is indicated using a *green circle*, and the PM curves are plotted using *colored lines*—APM (*blue*) and BPM (*red*). *Left and center column plots* are using 2-norm in (*A*, *B*) and ($\log (A),B$) space, respectively, while the *right column plots* are using log-norm

We start by looking at the simplest curve fitting involving an exponential function (1). Assuming that the “observed” data has no noise and that the number of observations is infinite, we have $\overline{\sigma }=\bar{A}e^{\bar{B}\epsilon }\;\forall \epsilon \in [0,E]$. Here, $E>0$ defines the maximum value of strain $\epsilon$ at which the stress has been observed. Our model (1) needs to be fit to $\overline{\sigma }$ and, thereby, determine the optimum parameters *A* and *B*.

As a first step, we simply take our functional as the 2-norm of the difference between the observed data and model: $\mathcal {F}={||} \sigma - \overline{\sigma }{||}_2=\int \limits _0^E \left( Ae^{B\epsilon }-\bar{A}e^{\bar{B}\epsilon } \right) ^2\,\mathrm{d}\epsilon$, which can be evaluated analytically (27) (details of all analytical derivations are in “Details of the analytical derivations”). Clearly, $\mathcal {F}=0$ at $A=\bar{A}$ and $B=\bar{B}$, and $\mathcal {F}>0$ everywhere else. Thus, it has a global minimum at $(\bar{A},\bar{B})$, which corresponds to the true solution. Also, it can be easily verified that there are no other local minima in this functional. That is, $(\bar{A},\bar{B})$ is the unique global minimum and $\mathcal {F}$ is convex everywhere. Furthermore, this is a highly nonlinear functional, and its contour plot contains a long narrow valley similar to that observed in the inverse model of a bioprosthetic valve (Figs. 2, 6a). Hence, interestingly, curve fitting of a simple exponential function reproduces the behavior seen for a complex inverse modeling problem.

For this function, the APM and BPM curves can also be determined in closed form (29). These curves lie very close together in the valley of the functional (Fig. 6a). Since a point in the parameter space where both derivatives are zero represents the global minimum, the proximity of the two PM curves implies that both derivatives are *approximately zero* in the whole valley region. That is why, even though the functional is convex, parameters along the valley produce a similar response $\sigma (\epsilon )$, as observed previously (Fig. 2).

Based upon elementary algebra, for fitting an exponential function, it is significantly easier if the function is linearized by taking a logarithm before the 2-norm. That is, $\mathcal {F}={||} \log (\sigma ) - \log (\overline{\sigma }) {||}_2$. This leads to a quadratic functional in $\log (A)$ and *B* (31), where both APM and BPM curves are straight lines (33) (Fig. 6c). Clearly, this norm also satisfies the condition of a unique global minimum at $(\bar{A},\bar{B})$. More importantly, the two PM curves are not close anymore and are visually distinct (Fig. 6c). This is reflected in the functional shape as an absence of a valley.

Since $\sigma >0$ and $\overline{\sigma }>0$ for this model for all strain values, the norm $\mathcal {F}={||} \log (\sigma ) - \log (\overline{\sigma }) {||}_2$ is equivalent to the log-norm (12). There is one subtle difference between the 2-norm and log-norm functionals: the latter is defined in a transformed parameter space of $(\log (A),B)$ instead of (*A*, *B*). Therefore, in order to objectively compare the two norms, we look at the 2-norm in the $(\log (A),B)$ parameter space (Fig. 6b). In this case, the APM and BPM curves are still close together; however, the valley becomes relatively straight.

We extend these ideas to function (3), which is a better representation of stress–strain relation. With 2-norm, we obtain a similar curved valley in the (*A*, *B*) space (Fig. 6d), where the PM curves are even closer together. In the $(\log (A),B)$ space using 2-norm, the PM curves and the valley are more straight (Fig. 6e). For the log-norm, modified log function (13) has to be used, since $\sigma$ goes to zero for $\epsilon =0$ making its log undefined. For (3) with log-norm, it is not possible to obtain analytical expressions for the functional and PM curves. Thus, these were determined numerically using representative values of $\bar{A}=0.02$ and $\bar{B}=45$. Again, we observe disappearance of the valley, and the two PM curves become distinct for $B\gtrapprox 10$ (Fig. 6f).

We perform similar analysis for multi-dimensional constitutive laws using DC curve fitting. In this case, the functional and PM curves cannot be determined analytically for either of the norms, so numerical calculations were performed using SM and GOH models for the same representative parameter values ($\bar{A}=0.02$ and $\bar{B}=45$). The resulting functionals behave very similar to the one-dimensional problems. The 2-norm has a similar valley which is curved in the (*A*, *B*) space and practically straight in the $(\log (A),B)$ space, and the PM curves are extremely close in both cases (Fig. 6g–l)^1^. Using the log-norm, the PM curves become distinct and no valley is observed (Fig. 6i, l). Lastly, a small difference can be noticed between the 2-norm functionals for two constitutive laws: the PM curves for SM model are closer together as compared to the GOH model.

**Fig. 7:**
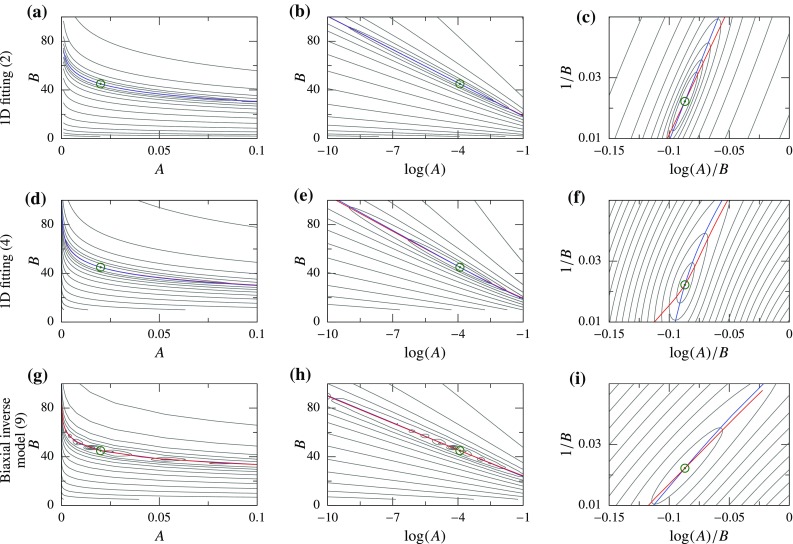
Functional for force-controlled (FC) cases plotted as a *contour*; global minimum is indicated using a *green circle*, and the PM curves are plotted using *colored lines*—APM (*blue*) and BPM (*red*). All functionals are evaluated using 2-norm but plotted in different parameter spaces; from *left to right column*: (*A*, *B*), ($\log (A),B$), and ($\log (A)/B,1/B$) space, respectively

#### Force-controlled cases

Similar to the analysis of DC cases, we start with the simplest FC problem: the inverse of an exponential function (2). We assume that the observed data is of the same form without any noise, i.e., $\overline{\epsilon }(\sigma )=\left( \log (\sigma /\bar{A}) \right) /\bar{B}$, and that the observations were made continuously in the range $\sigma \in [1,\Sigma ]$. Here, $\Sigma$ is the maximum stress at which strain was observed. In order to fit our model to the observed data, as a first step, we take the 2-norm functional: $\mathcal {F}={||} \epsilon - \overline{\epsilon }{||}_2$. This can be evaluated analytically and results in a rational functional (35). Similar to the DC case, a long and narrow valley is observed (Fig. 7a). Here also, we find that the PM curves (37) and (39) essentially overlap. Furthermore, the valley and PM curves become relatively straight in the $(\log (A),B)$ space (Fig. 7b)—another similarity to the DC case.

**Fig. 8:**
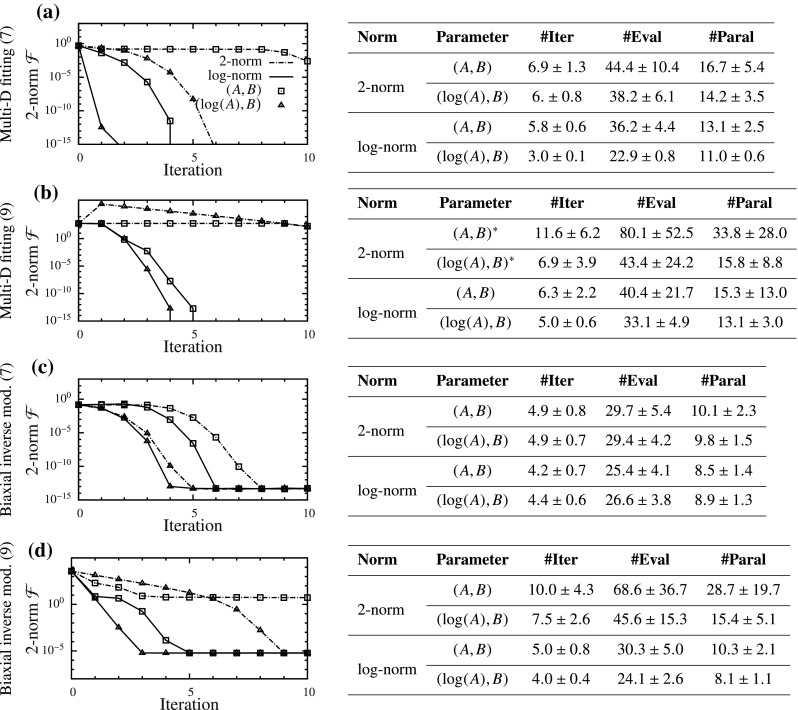
Parameter estimation results for four displacement-controlled (DC) cases with one representative example of convergence each (*left*), and statistics with starting points spanning the parameter space (*right*) summarized as mean ± SD (*iterations from some starting points did not converge)

However, unlike the DC case, changing the norm to log-norm, or any other norm, does not make the model linear, and the valley shape of the functional persists. Instead, if the function (2) is rewritten as:

19 $$\begin{aligned} \epsilon (\sigma )=\frac{1}{B}\log (\sigma )-\frac{\log (A)}{B}, \end{aligned}$$

it is easy to see that defining a new pair of parameters

20a $$\begin{aligned} \alpha&= \frac{\log (A)}{B} \text { and}\end{aligned}$$

20b $$\begin{aligned} \beta&= \frac{1}{B}, \end{aligned}$$

makes the strain $\epsilon (\sigma )=\beta \log (\sigma )-\alpha$ linear in parameters $\alpha$ and $\beta$. Hence, the parameter estimation problem using 2-norm becomes a linear least-square problem. In other words, the transformation of parameters from *A*, *B* to $\alpha ,\beta$ makes the 2-norm functional $\mathcal {F}(\alpha ,\beta )={||} \epsilon (\alpha ,\beta ) - \overline{\epsilon } {||}_2$ quadratic (Fig. 7c). Concomitantly, the PM curves (36) and (38) become straight lines that are distinct from each other.

This idea is extended to function (4), where, even though the transformation does not make the function exactly linear, it exhibits a similar behavior in the functional shape and PM curves (Fig. 7d–f). That is, in the original parameter space (*A*, *B*), the 2-norm functional has a narrow and curved valley. Transforming the parameter space to $(\log (A),B)$, the valley becomes straight, but the PM curves remain close together. Lastly, using the transformation $(\log (A)/B,1/B)$, the valley ceases to exist, and the PM curves become distinctly different.

For the FC cases, curve fitting cannot be performed for multi-dimensional constitutive models. Instead, we calculate the functional and PM curves for biaxial inverse model using SM. Even this multi-dimensional problem, which involves a highly complex stress–strain function, behaves very similar to the 1D problems. With 2-norm, we obtain a narrow and curved valley in the (*A*, *B*) space (Fig. 7g), which becomes straight in the $(\log (A),B)$ space (Fig. 7h). In both of these cases the PM curves remain close together. However, in the $(\log (A)/B,1/B)$ space we do not see any valley and the PM curves become distinct (Fig. 7i). The functional shape and PM curves can be useful indicators for the parameter estimation, as we see next.

**Fig. 9:**
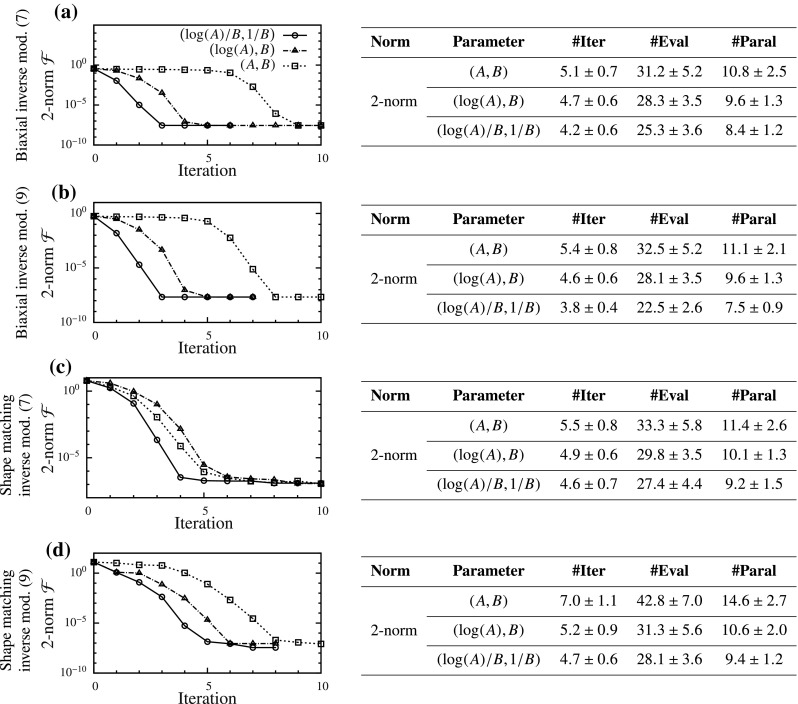
Parameter estimation results for four force-controlled (FC) problems with one representative example of convergence each (*left*), and statistics with starting points spanning the parameter space (*right*) summarized as mean ± SD

### Parameter estimation

Based upon the analysis, we compare the following four formulations for the DC cases:

1. 2-norm in $\left( A,B\right)$ parameter space
2. 2-norm in $\left( \log (A),B\right)$ parameter space
3. log-norm in $\left( A,B\right)$ parameter space
4. log-norm in $\left( \log (A),B\right)$ parameter space

On the other hand, for FC cases, we compare the following three formulations—all with 2-norm:

1. $$\left( A,B\right) $$ parameter space
2. $$\left( \log (A),B\right) $$ parameter space
3. $$\left( \log (A)/B,1/B\right) $$ parameter space

#### Displacement-controlled cases

All four DC cases tested using the algorithm with four formulations were summarized (Fig. 8). The 2-norm functional was plotted versus iterations for one representative starting point, and average statistics for all starting points were tabulated. There was a consistent pattern that the number of iterations needed to converge decreased as we changed from formulation 1–4, with log-norm in $(\log (A),B)$ space producing the best results. The decrease was modest in some cases (Fig. 8c), while it was as high as 60% in others (Fig. 8a, d). Furthermore, convergence was slower with SM model (Fig. 8b, d) compared to the GOH model (Fig. 8a, c). In fact, the iterations for SM curve fitting with 2-norm diverged for a few initial guesses. Secondly, we also found a clear decrease in the SD of number of iterations as we go from case 1–4. This shows that by using log-norm and $(\log (A),B)$ space, the problem becomes uniformly converging.

A couple of worthwhile remarks about the implementation of the proposed algorithm: it was observed that when the minimization routine uses a finite element solver for model calculation (FEBio in this study), care must be taken with the floating point precision of input and output. If input and output are in ASCII format, severe truncation errors can be introduced into the minimization routine. These truncation errors can adversely affect the proposed minimization algorithm, especially with small TOL and $\delta$ values. This can be alleviated to some extent by increasing the precision of floating point numbers in the input and output file format. Effect of this truncation error can be seen in convergence plots of inverse problems (Fig. 8c, d), where the functional did not decrease beyond a value. This minimum value did not change with the formulation and thus was only dependent on the problem setup. Curve fitting problems did not show such a minimum (Fig. 8a, b). Secondly, the Gauss–Newton equation (16) sometimes gives large step sizes ($\Delta \mathbf {c}$), such that the current guess $\mathbf {c}$ may go outside the physiological range. This can adversely affect the line search and calculation of *f* may fail occasionally. This is why the condition of function calculation being successful was added in the algorithm (Algorithm 1, line 10).

#### Force-controlled cases

For all FC cases, the parameter estimation improved as we changed from formulation 1–3 (Fig. 9). The formulation with 2-norm and $(\log (A)/B,1/B)$ space performed the best in all problems. However, the improvements in convergence speed were smaller than those in DC cases, with the maximum reduction in iterations required being 35% (Fig. 9d). The SD also decreased as we went from formulation 1–3, showing that problem converged more uniformly. Lastly, the GOH model (Fig. 9a, c) performed marginally better than the SM (Fig. 9b, d) in the (*A*, *B*) space. However, the improvements due to new formulations were smaller for GOH model than those for the SM. Since all of the problems were inverse models that involved interfacing with FEBio, functionals did not decrease beyond a minimum value (similar to the DC inverse models).

### Noise

**Fig. 10:**
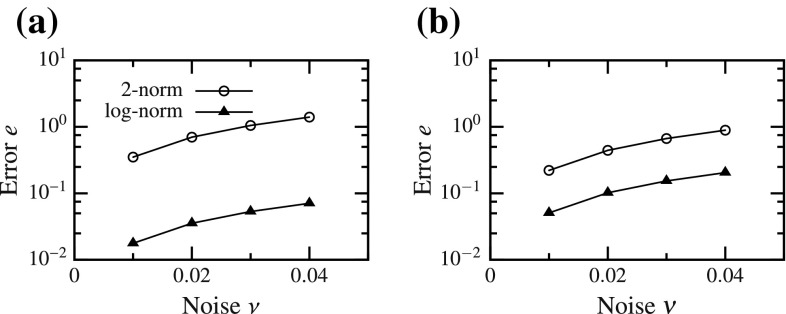
Change in error as a function of random noise introduced into the target vector for DC cases. **a** structural model and **b** GOH model

When noise was introduced into the observed data, the global minimum of the functional moved away from the true solution for multi-dimensional curve fitting with both SM (9) and GOH (7) models. As the level of noise ($\nu$) was increased, the error in estimated parameters also increased. However, for both models, the increase in error was significantly lower using the log-norm as compared to the 2-norm (Fig. 10). Change of parameter space between (*A*, *B*) and $(\log (A),B)$ had no effect on the error. Since norm was not changed in the FC cases, the variation of error as a function of noise remained the same irrespective of the parameter space used (results skipped for brevity).

### Heterogeneous model

**Fig. 11:**
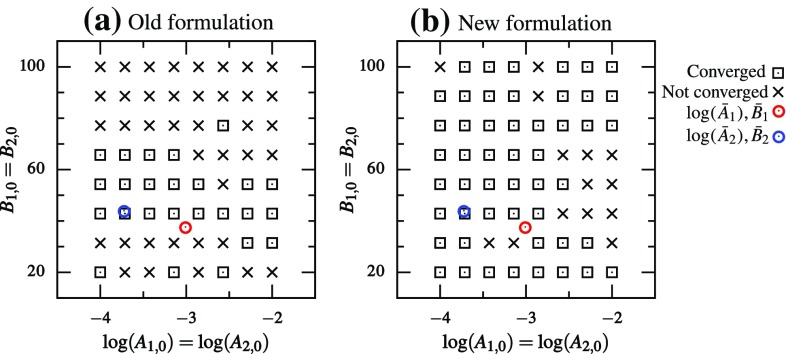
Plot showing whether the parameter estimation converged to the true parameters for DC case with different starting guesses using **a** 2-norm and (*A*, *B*) parameter space, and **b** log-norm and $(\log (A),B)$ parameter space. Each *cross/square* is the starting guess, and *circles* indicate true parameters

The true parameters ($\bar{A}_1,\bar{B}_1,\bar{A}_2,\bar{B}_2$) were chosen from the valley region of the 2-norm functional (i.e., on either APM or BPM curve—the two curves essentially overlapping). This led to a system where the two materials produced a similar response and were difficult to differentiate. Only the convergence/non-convergence^2^ was recorded and compared between the two formulations that performed best and worst in the homogeneous parameters estimation. That is, for the DC case, (a) 2-norm with (*A*, *B*) space and (b) log-norm with $(\log (A),B)$ space were used. The results from various starting points for the DC case showed a clear difference between the two formulations (Fig. 11). Even though the second formulation did not give perfect results (50 out of the 64 initial guesses led to convergence), it performed significantly better compared to the first formulation (only 25 out of the 64 initial guesses led to convergence).

**Fig. 12:**
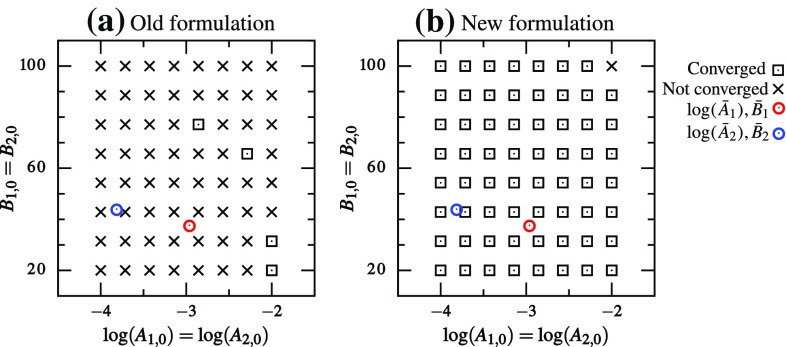
Plot showing whether the parameter estimation converged to the true parameters for FC case with different starting guesses using **a** 2-norm and (*A*, *B*) parameter space and **b** 2-norm and $(\log (A)/B,1/B)$ parameter space. Each *cross/square* is the starting guess, and *circles* indicate true parameters

In the FC case, two formulations with (a) (*A*, *B*) space and (b) $(\log (A)/B,1/B)$ space were used, both with 2-norm functional. The improvement in this case was much more significant (Fig. 12). Only 4 out of the 64 starting guesses resulted in convergence using the original parameters, whereas 63 out of 64 starting guesses resulted in convergence using the transformed parameters. Furthermore, the DC and FC cases had closely equivalent boundary conditions and true parameters $(\bar{A}_1,\bar{B}_1,\bar{A}_2,\bar{B}_2)$. Therefore, it is worthwhile comparing their performance. In the standard formulation (2-norm and (*A*, *B*) space), the DC case was better conditioned than the FC case and led to convergence more often (Figs. 11a, 12a). However, in the modified formulation, FC case significantly outperformed the DC case (Figs. 11b, 12b).

**Fig. 13:**
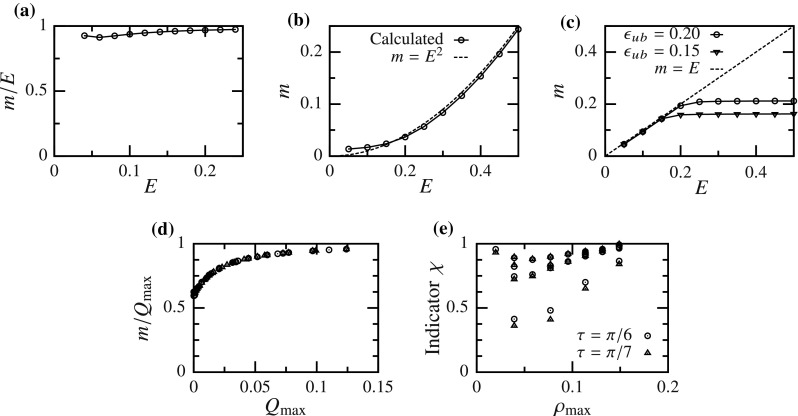
Variation of the slope *m* of the valley in functional landscape for various models—**a–c** for one-dimensional models (3), (5) and (6), respectively, and **d** and **e** for multi-dimensional GOH (7) and structural (9) models, respectively

### Parameter comparison

The valley in the functional is a region of high covariance between the two parameters *A* and *B*, and parameters along this valley produce similar stress–strain response while those across it do not (Fig. 2). Therefore, knowing the equation of the valley will greatly facilitate parameter comparison. Using $(\log (A),B)$ parameter space with 2-norm, a straight valley was observed in the functional for all problems (Figs. 6, 7). Therefore, as a generalization, we approximate the equation of the valley as a straight line with slope *m* passing through the optimal parameters $(\bar{A},\bar{B})$, i.e.,

21 $$\begin{aligned} \log (A/\bar{A})+m(B-\bar{B})=0. \end{aligned}$$

However, the dependence of slope *m* on a problem presents a challenge, as one would have to calculate it numerically for each specific setup. Thus, we determined the relation between the slope of valley and various parameters for several 1D and multi-dimensional problems, with the aim of establishing a general criterion on how to approximate the slope without explicitly calculating it.

In all problems, we numerically verified that the slope of the valley did not depend on the true parameters $(\bar{A},\bar{B})$ (within the physiological range). In 1D, for exponential function (3), the slope was well approximated by the maximum value of strain: $m\approx E$ (Fig. 13a). This can also be confirmed by manipulating the analytical expression of PM curves (29). For exponential function with higher nonlinearity (5), $m\approx E^2$ (Fig. 13b). For truncated exponential function (6), we found that $m\approx \epsilon$ if $\epsilon \le \epsilon _\mathrm{ub}$ and $m\approx \epsilon _\mathrm{ub}$ otherwise (Fig. 13c). Thus, in general, the slope of the valley is well approximated by the highest coefficient of *B* in the exponential.

Similarly, for multi-dimensional GOH model (7), slope was approximately equal to the maximum value of the exponent *Q*: $m\approx Q_{\text {max}}$ (Fig. 13d). For the structural model (9), stress is an integral of the exponential function with varying exponent $B(\varvec{N}\cdot \mathbf {E}\cdot \varvec{N})$, where $\rho (\varvec{N})=\varvec{N}\cdot \mathbf {E}\cdot \varvec{N}$ is the extension in direction $\varvec{N}$. Since there is not a single value of the exponent at the *maximum load*, we denote the maximum and minimum coefficients of *B* as $\rho _{\max }$ and $\rho _{\min }$, respectively. Next, we assume that the slope *m* lies between these two values, i.e., $\rho _{\min } \le m \le \rho _{\max }$. In order to verify this assumption, we define an indicator

22 $$\begin{aligned} \chi =\left( {\frac{m}{\rho _{\max }}-\frac{\rho _{\min }}{\rho _{\max }}}\right) /\left( {1-\frac{\rho _{\min }}{\rho _{\max }}}\right) . \end{aligned}$$

We found $0 \le \chi \le 1$ (Fig. 13e) for all cases, thus proving our assumption. Moreover, for most of the cases, the slope was well approximated by the maximum extension $\rho _{\max }$ (i.e., $\chi \approx 1$). Only in the case when the principal stretches differed significantly *and* the maximum stretch was not aligned with the material direction, $\chi \not \approx 1$. Thus, in most of the situations, it is possible to approximate the valley’s slope simply from the maximum applied strain and tissue microstructure.

## Discussion

### Effect of exponential function

Soft tissue’s mechanical response is characterized by high nonlinearity, anisotropy and non-homogeneity. The nonlinearity feature is represented as an exponential function in many of the stress–strain relationships (Maurel et al. 1998, chap. 4). Motivated by some observations from our previous work (Aggarwal and Sacks 2016), we aimed to analyze the effect of this high nonlinearity on elastic parameters. We found that it is a fundamental feature of the exponential function that leads to a narrow valley in the functional, irrespective of the complexity of the problem setup or constitutive law details. Furthermore, we categorized all the problems into DC and FC cases and found a similar functional shape for both problems. That is, the functionals contained a characteristic valley shape which was related to the proximity of two PM curves (Figs. 6, 7). This functional valley resulted in an ill-conditioned system, high covariance between the two elastic parameters, slow convergence during parameter estimation, and misleading conclusions in parameter comparison.

### Significance of presented results

In order to solve these challenges, we drastically simplified the problem by looking at an exponential function (1). Although this may seem like an oversimplification at first, the function reproduced the behavior observed for soft tissues. Thus, extending the ideas from elementary algebra, we found two different solutions for the two categories of problems. For DC cases, we found that using log-norm and $(\log (A),B)$ space made the parameter estimation close to linear and improved the condition of the problem. This was graphically depicted using two PM curves and separation between them. On the other hand, for FC cases, we found that by keeping the 2-norm but using $\left( \log (A)/B,1/B \right)$ space, the problem’s condition was significantly improved. These qualitative changes were consistently observed irrespective of the specific details of the problem (Figs. 6, 7). The new formulations led to an increase in the convergence speed for all problems considered (Figs. 8, 9). The increase in convergence was significant in some problems, while limited in others. In general, the improvements were smaller for the FC cases compared to those in DC cases. Also, the closeness of two PM curves was found to be related to the convergence speed, thus proving to be a useful indicator.

Secondly, we found that using 2-norm and $(\log (A),B)$ space, the functional contained a straight valley for all problems studied here. The slope of the valley was found to be well approximated by the highest coefficient of *B* for most of the problems (Fig. 13). Therefore, it is possible to estimate the slope of the valley in the 2-norm functional for a specific problem based upon the applied strains and microstructural properties, without performing any minimization or parameter estimation. It was established that changing parameters along the valley minimally affects the overall stress–strain response, whereas changing the parameters across the valley dramatically varies the response (Fig. 2).

#### Proposed modifications

Based upon these results we propose to use log-norm for DC cases and 2-norm for FC cases. Furthermore, we propose the use of $(\log (A),B)$ space and $(\log (A)/B,1/B)$ space for DC and FC cases, respectively. We note that for the second proposition, since *A* has the units of stress, taking its log would be physically nonstandard. Therefore, we propose a modified form of the constitutive laws. Instead of $\sigma ({A},B,\epsilon )\sim Ae^{B\epsilon }$, we propose to use a form

23 $$\begin{aligned} \sigma (\hat{A},B,\epsilon )\sim \eta e^{\hat{A}}e^{B\epsilon }, \end{aligned}$$

where $\eta$ is a known and specified number with units of stress (e.g., stiffness of collagen fibrils). This modification, in addition to making the parameter $\hat{A}$ dimensionless, can also be used to scale $\hat{A}$ so that its value is in the same range as *B*, thus improving the numerical conditioning of the problem. Another benefit of using this modified form is that now $\hat{A}\in \mathbb {R}$ is not restricted to be positive as *A* was, thus reducing the constraints on the parameter estimation problem.

**Fig. 14:**
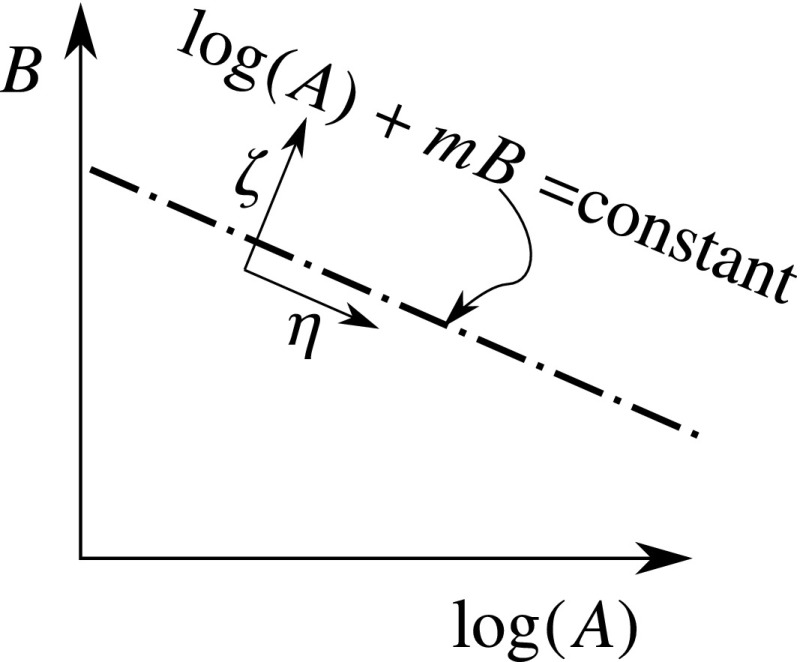
Definition of problem-specific parameters $\zeta$ and $\eta$ to facilitate parameter comparison

Using the valley’s slope in the functional space, which can be approximated for a given problem setup, we define a new set of parameters (Fig. 14)

24a $$\begin{aligned} \zeta&=\log (A)+mB \text { and}\end{aligned}$$

24b $$\begin{aligned} \eta&=m\log (A)-B. \end{aligned}$$

These parameters may be used to rationally compare elastic parameters of tissue samples, so as to truly represent the difference between their resulting stress–strain relationship. We call $\zeta$ the “major parameter” as it varies the stress–strain relation significantly, and $\eta$ the “minor parameter” as its effect on the stress–strain relation is minimal.

**Fig. 15:**
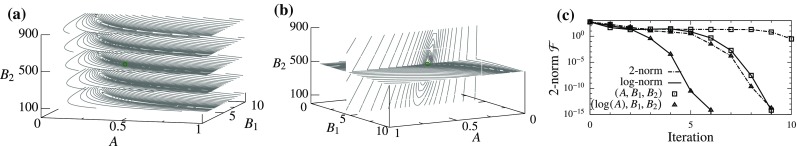
Results for model by May-Newman and Yin (1998) with three parameters: **a** contour of the 2-norm shows valleys in the $AB_1$-planes, and **b** valleys in $AB_1$- and $AB_2$-planes interact to provide a complex 3D functional space [*green circle* denotes global minimum: $(\bar{A},\bar{B}_1,\bar{B}_2)=(0.4,4.5,500)$]; **c** the proposed modification significantly improves the convergence speed

#### Faster convergence

One of the primary effects of the new formulations was the change in the functional shape (absence of a valley), which led to faster convergence of the parameter estimation (Figs. 8, 9). Although faster convergence may seem inconsequential for curve fitting procedures, it can save substantial computational time for inverse models where each iteration may take minutes or hours to compute. This is becoming more important as inverse models are increasingly being recognized as the most appropriate way of characterizing mechanics of soft tissues (Rausch et al. 2013; Zhang et al. 2015). Curve fitting assumes uniform microstructural properties and uniform strain, which usually do not hold true for soft tissues because of their heterogeneity. Therefore, inverse modeling is seen as a more accurate tool, and improved parameter estimation has the potential for making them more widely usable and, possibly, suitable for bedside computations.

#### More than improved speed

Although faster convergence is an important result, the changes in the functional shape with the proposed formulations had other implications as well. We found that the parameter estimation becomes more robust with respect to noise when using log-norm for DC cases (Fig. 10). However, the effect of noise only depends on the norm and not the parameter space used. Thus, no improvement was observed for FC cases with the new formulation.

Interaction between parameters of multiple materials in a heterogeneous system may lead to the formation of local minima, even with simple constitutive models. Here, a problem with two sets of elastic properties was tested. We found that when using the modified formulations, chances of getting trapped in a local minima were significantly reduced (Figs. 11, 12). Furthermore, we also found that FC case performed better than the DC case in heterogeneous inverse model. Thus, if there is an option to choose between FC and DC settings for a study, the former might provide more robust results using the new formulation.

#### Extension to multiple parameters

In this work, we used two forms of the constitutive laws; however, the results presented are expected to be general and applicable to other forms involving an exponential function. The analysis could also be extended to the case of multiple parameters, although with additional complexity. For instance, the model by May-Newman and Yin (1998) describes strain energy density $\Psi (\mathbf {F})=A\left( e^Q-1 \right)$, where $Q=B_1\left( I_1 -3\right) ^2 + B_2 \left( \sqrt{I_4} -1\right) ^4$, and thus has three elastic parameters $(A,B_1,B_2)$. In this case, in the three-dimensional parameter space, we obtain a valley in the $AB_1$-plane for each fixed $B_2$, and vice versa in $AB_2$-planes (Fig. 15a, b). Despite these differences, the proposed modification leads to an improved convergence (Fig. 15c). Similarly, in models with multiple exponential terms (Holzapfel et al. 2000; Holzapfel and Ogden 2009), each term will lead to a valley while other terms are held fixed. Interaction of different valleys may lead to a complex behavior. For example, the heterogeneous model presented in this study is qualitatively similar to having multiple fiber families in the constitutive model (Holzapfel et al. 2000), which led to the formation of local minima. Further analysis of such interactions among different terms is necessary, but is beyond the scope of present manuscript. Similar convexity analysis is also required for constitutive models with a large number of parameters, e.g., Fung’s law (Labrosse et al. 2016).

### General implications

We chose a minimization algorithm based upon Gauss–Newton and line search methods (Algorithm 1). However, the proposed modifications are expected to benefit all minimization routines for this class of problems. This is because of the fundamental changes in the functional shape that should positively affect the convergence properties irrespective of the algorithm used. In fact, many of the other methods, such as gradient-based Levenberg–Marquardt and evolution-based genetic algorithm (Lee et al. 2014; Labrosse et al. 2016), perform poorly in the presence of narrow valleys in the functional (Nocedal and Wright 2006). Therefore, the improvements from proposed modifications may be even greater for those methods.

Covariance between parameters plays an important role in the design of experiments. Here, we found that the slope of high covariance region is related to the maximum strain applied. That is, applying a higher strain leads to a more horizontal valley, which represents a lower covariance between the two parameters. The analysis presented here could be extended to find optimum points of observation so as to establish maximum confidence in the estimated parameters. This is extremely important for non-convex problems, since the solution could depend upon initial guess due to the presence of local minima (Abbasi et al. 2016).

The proposed modifications are simpler for FC cases since it conserves the use of 2-norm. We also observed that the FC case performed better than the DC case in the heterogeneous parameter estimation, which indicates that FC setup may be better suited for inverse modeling. For all these reasons, we consider this work as the discovery of a new direction in soft tissue biomechanics that would lead to multiple future studies as discussed next.

### Limitations and future work

In this study, we considered only elastic or hyperelastic problems, but similar analysis could be done for time-dependent models (such as viscoelastic), which are widely used for soft tissues. Furthermore, we restricted to only two parameters in order to facilitate the analysis and visualization of the functional shape. Extending the work to multiple parameters will be of significant value and will be completed in a future study. Although we only used planar tissues as examples here, the ideas are expected to be applicable to thick tissues, e.g., myocardium. Taking a log-norm assumes that all the stresses are positive, which is reasonable since exponential laws should not produce negative values. However, this may not hold true in general, especially for shear stresses. This issue was not addressed here and will be investigated in the future.

Our treatment of the heterogeneous media was not exhaustive. Examples were used to only demonstrate the advantages of the proposed modifications. As heterogeneous inverse problems become exponentially complex as the number of materials increase, we limited the current study to only two material types. Although the proposed modifications led to improved results, they were still not perfect as occasional non-convergence was observed. A more thorough analysis of these factors will be performed in the future, especially when noise is present in a heterogeneous system.

The minimization algorithm proposed here also could be improved, e.g., by using a better line search method, using forward difference instead of central difference for the gradient, carefully chosen $\delta$ value, etc. Hence, the focus here was not on designing the best minimization algorithm, but to use the insight on fundamental features that will benefit all minimization methods. Similarly, innovative ideas could be used to improve the convergence for specific problems. For example, using $(\zeta ,\eta )$ parameter space and minimizing the functional with respect to only the major parameter $\zeta$ first, followed by complete minimization could provide significant speedup.

In addition to parameter estimation, the proposed modifications could also improve the material and shape optimization of bioprosthetics (Fan et al. 2013). These ideas could also be applied to other techniques, such as elasticity imaging (Oberai et al. 2009) and deformable image registration (Veress et al. 2005). Lastly, the non-elastic parameters were assumed to be known, which is true for several tissues, such as heart (Nielsen et al. 1991), aorta (Haskett et al. 2010) and aortic valve (Aggarwal et al. 2014). In the future, we will study the case when non-elastic parameters also need to be determined from inverse models, e.g., for pathological tissues.

## Conclusion

In this study, the aim was to elucidate some of the issues related to soft tissue constitutive laws observed in our last study. We found that these features are fundamental to the exponential function, which is commonly used in soft tissue mechanics. By simplifying the problem and using elementary algebra, we proposed solutions that showed improved convergence and robustness with respect to noise. More importantly, the modified formulations were found to be less susceptible to being trapped in a local minima for heterogeneous problems. We also proposed modified parameters that will facilitate a rational comparison of elastic parameters. The insight obtained in this study will be used in the future to significantly improve inverse models and provide higher confidence in the elastic parameters used for soft tissue biomechanical studies.

## Appendix

### Details of biaxial simulation

Biaxial stretching of a 1 $\times$ 1 cm specimen of thin soft tissue with a uniform thickness of 0.46 mm was simulated. Since markers are usually applied in the center one third where deformation is assumed to be approximately homogeneous, 1/3 $\times$ 1/3 cm tissue was modeled using Reissner–Mindlin thin shell elements. A 40 $\times$ 40 quadrilateral element mesh was used with the tissue in *xy*-plane (Fig. 3). The fiber direction was predominantly in the *x*-direction ($\omega \approx 0$) and fiber dispersion $\tau$ was approximately $35^\circ$, with small perturbations which made it more appropriate to be solved using inverse modeling rather than by curve fitting. The neo-Hookean matrix stiffness in (8) was set at $\lambda =172.84\,\hbox {kPa}$ and $\upmu =74.1\,\hbox {kPa}$ (corresponding to a linearized Young’s modulus of 200 kPa and Poisson’s ratio of 0.35). The lower and left edges were fixed in the *yz* and *xz* directions, respectively, and free to deform in the third direction (Fig. 3). For DC case, uniform displacement was applied on the right and upper edge of the tissue so as to generate a maximum strain of 13 and 8% in *x*- and *y*-direction, respectively. For FC case, a uniform force was applied on the right and upper edges in the normal direction: *x*-force of 0.4 N on the right edge and *y*-force of 0.26 N on the upper edge. The applied force or deformation was linearly increased at each quasi-time step, and system was solved for static equilibrium. The resulting normal reaction force or deformation was averaged at right and upper edges at 151 uniformly spaced steps between unloaded and maximally loaded states (both inclusive).

### Details of tissue pressurization simulation

A semilunar-shaped single tissue representing a bioprosthetic valve leaflet with an area of 2.3 cm$^2$ and thickness of 0.38 mm was simulated using Reissner–Mindlin thin shell elements. The sample geometry was meshed using 2789 quadrilateral elements with constant thickness (Betsch et al. 1996). Displacement was interpolated using bilinear shape functions, and stresses were integrated through the thickness using three point Gauss quadrature rule to obtain bending moments. The fibers were aligned approximately in the diagonal direction with a fiber dispersion $\tau \approx 35^\circ$ and relatively small perturbations over the sample. Contact of the sample with other leaflets was modeled using two idealized rigid planes placed at $\pm 60^\circ$ with respect to the sample’s plane of symmetry (Fig. 4).

The neo-Hookean matrix stiffness in (8) was set at $\lambda =172.84\,\hbox {kPa}$ and $\upmu =74.1\,\hbox {kPa}$ (corresponding to a linearized Young’s modulus of 200 kPa and Poisson’s ratio of 0.35). A uniform normal pressure was applied on the tissue with a maximum value of 120 mm of Hg. The contact was solved using augmented Lagrange method as pressure was linearly increased. At each load step, static equilibrium equations were solved to obtain the deformed shape of the tissue sample, which was recorded at four uniformly spaced points between unloaded and maximally loaded states (both inclusive). The leaflet shape was compared using 129 virtual markers, where $L_2$-norm of the closest-point projection onto the deformed mesh was the objective function, as defined previously (Aggarwal and Sacks 2016).

### Details of the analytical derivations

#### For $Ae^{B\epsilon }$ and $A\left( e^{B\epsilon }-1\right)$ using 2-norm

For both functions (1) and (3), we use a common representation $\sigma (\epsilon )=Ah(B\epsilon )$. Thus, $h(B\epsilon )=e^{B\epsilon }$ corresponds to (1) and $h(B\epsilon )=e^{B\epsilon }-1$ corresponds to (3). Using 2-norm, we have

25 $$\begin{aligned} \mathcal {F} (A,B) = \int \limits _0^E \left[ Ah(B,\epsilon ) - \bar{A}h(\bar{B},\epsilon ) \right] ^2 \mathrm{d}\epsilon . \end{aligned}$$

If we denote

26 $$\begin{aligned} \int \limits _0^E h(B,\epsilon )h(\bar{B},\epsilon ) \mathrm{d}\epsilon = g(B,\bar{B}), \end{aligned}$$

the functional can be written in a simplified form

27 $$\begin{aligned} \mathcal {F} (A,B) = A^2g(B,B) + \bar{A}^2 g(\bar{B},\bar{B}) -2A\bar{A}g(B,\bar{B}). \end{aligned}$$

The gradient of functional w.r.t. *A* and *B* is

28 $$\begin{aligned} \nabla \mathcal {F} = \left[ \begin{array}{ccc} 2Ag(B,B)-2\bar{A}g(B,\bar{B}) \\ 2A^2g'(B,B) - 2A\bar{A}g'(B,\bar{B}) \end{array} \right] ^T, \end{aligned}$$

where $'$ denotes differentiation w.r.t. *B*. The two PM curves are obtained by condition $\nabla \mathcal {F}=0$:

29a $$\begin{aligned} \text {APM: }Ag(B,B)&= \bar{A}g(B,\bar{B}) \text { and}\end{aligned}$$

29b $$\begin{aligned} \text {BPM: }Ag'(B,B)&= \bar{A}g'(B,\bar{B}). \end{aligned}$$

Thus, the equations of two curves are similar except for the replacement of *g*(*B*) with $g'(B)$.

#### For $Ae^{B\epsilon }$ using log-norm

For function (1) and log-norm, expanding the functional $\mathcal {F}(A,B)=\int \Vert \log (\sigma (A,B,\epsilon ))-\log (\overline{\sigma }(\bar{A},\bar{B},\epsilon )) \Vert ^2\,\mathrm{d}\epsilon$, we have

30 $$\begin{aligned} \mathcal {F}(A,B)= \int \limits _0^{E} \left[ \left( \log (A)-\log (\bar{A})\right) + (B-\bar{B})\epsilon \right] ^2\, \mathrm{d}\epsilon . \end{aligned}$$

If we denote $\alpha =\log (A), \overline{\alpha }=\log (\bar{A}), \beta =B$ and $\overline{\beta }=\bar{B}$, we obtain

31 $$\begin{aligned} \mathcal {F}(\alpha ,\beta ){=} E\left( \alpha -\overline{\alpha }\right) ^2 + \frac{E^3}{3}\left( \beta -\overline{\beta }\right) ^2 {+} E^2\left( \alpha -\overline{\alpha }\right) \left( \beta -\overline{\beta }\right) . \end{aligned}$$

The gradient of this functional w.r.t parameters $\alpha$ and $\beta$ is

32 $$\begin{aligned} \nabla \mathcal {F} = \left[ \begin{array}{ccc} 2E\left( \alpha -\overline{\alpha }\right) + E^2\left( \beta -\overline{\beta }\right) \;\\ E^2\left( \alpha -\overline{\alpha }\right) +\,2E^3/3\left( \beta -\overline{\beta }\right) \end{array} \right] ^T. \end{aligned}$$

The condition gradient $\nabla \mathcal {F}=0$ gives us equations of two PM curves, which, in this case, are straight lines,

33a $$\begin{aligned} \text {APM: }\left( \alpha -\overline{\alpha }\right)&+ \frac{E}{2}\left( \beta -\overline{\beta }\right) =0 \text { and}\end{aligned}$$

33b $$\begin{aligned} \text {BPM: }\left( \alpha -\overline{\alpha }\right)&+ \frac{2E}{3}\left( \beta -\overline{\beta }\right) =0. \end{aligned}$$

#### For $\left( \log (\sigma )-A\right) /B$ using 2-norm

For function (2), we use only the 2-norm. Therefore,

34a $$\begin{aligned}&\mathcal {F} (A,B) = \int \limits _1^\Sigma \left[ \epsilon (A,B,\sigma ) - \overline{\epsilon }(\bar{A},\bar{B},\sigma ) \right] ^2 \mathrm{d}\sigma \end{aligned}$$

34b $$\begin{aligned}&= \int \limits _1^\Sigma \left[ \log (\sigma )\left( \frac{1}{B}-\frac{1}{\bar{B}} \right) - \left( \frac{\log (A)}{B}-\frac{\log (\bar{A})}{\bar{B}} \right) \right] ^2 \mathrm{d}\sigma . \end{aligned}$$

Defining symbols $\alpha =\log (A)/{B}, \overline{\alpha }=\log (\bar{A})/{\bar{B}}, \beta =1/B$ and $\overline{\beta }=1/\bar{B}$, the functional can be simplified as:

35 $$\begin{aligned} \mathcal {F}(\alpha ,\beta )= \phi _1(\alpha -\overline{\alpha })^2 + \phi _2(\beta -\overline{\beta })^2 - 2\phi _3 (\alpha -\overline{\alpha })(\beta -\overline{\beta }) . \end{aligned}$$

Here, $\phi _1\!=\!\int \limits _1^\Sigma \mathrm d\sigma , \phi _2\!=\!\int \limits _1^\Sigma \left[ \log (\sigma ) \right] ^2 \mathrm d\sigma$, and $\phi _3\!=\!\int \limits _1^\Sigma \log (\sigma )\mathrm d\sigma$. We note that solving $\partial \mathcal {F}/\partial A=0, \partial \mathcal {F}/\partial \log (A)=0$, or $\partial \mathcal {F}/\partial \alpha =0$, all lead to the same equation. Or in other words, any of those equations could be used to determine APM curve. Therefore, for deriving the equation for APM curve, we use $\partial \mathcal {F}/\partial \alpha =0$ because of its simplicity:

36 $$\begin{aligned} \phi _3(\beta -\overline{\beta }) = \phi _1(\alpha -\overline{\alpha }), \end{aligned}$$

which is clearly a straight line in the $(\log (A)/B,1/B)$ space with slope $\phi _1/\phi _3$. This curve can be expressed in the original (*A*, *B*) coordinates as:

37 $$\begin{aligned} \phi _3\left( \frac{1}{B}-\frac{1}{\bar{B}}\right) = \phi _1\left( \frac{\log (A)}{B}-\frac{\log (\bar{A})}{\bar{B}}\right) . \end{aligned}$$

However, $\partial \mathcal {F}/\partial B=0$ and $\partial \mathcal {F}/\partial \beta =0$ are different curves. For BPM, its equation for the $(\log (A)/B,1/B)$ space is simply

38 $$\begin{aligned} \phi _2(\beta -\overline{\beta }) = \phi _3(\alpha -\overline{\alpha }) , \end{aligned}$$

which is also a straight line in $(\log (A)/B,1/B)$ space, although with a slope $\phi _3/\phi _2$. On the other hand, for (*A*, *B*) and $(\log (A),B)$ spaces, equation of BPM curve is given by

39 $$\begin{aligned} \frac{B}{\bar{B}} = \frac{\phi _2-\phi _3\log (A)-\phi _3\log (\bar{A})+\phi _1\log (A)\log (\bar{A})}{\phi _2-2\phi _3\log (A)+\phi _1\left[ \log (A)\right] ^2}. \end{aligned}$$

## Footnotes

1 The PM curves provide a visual and intuitive way of interpreting the effect of the modifications. The closeness of the PM curves could be quantified (e.g., by integrating the squared-difference between the two); however, that would not add much value to the analysis.

2 Both divergence and convergence to a wrong solution (i.e., local minima) are termed as non-convergence here.
